# Editorial: Widening participation and access to medicine as a career

**DOI:** 10.3389/fmed.2026.1867227

**Published:** 2026-06-16

**Authors:** Diantha Soemantri, Julie Willems, Gominda Ponnamperuma

**Affiliations:** 1 Faculty of Medicine Universitas Indonesia, Jakarta, Indonesia; 2 Faculty of Medicine, Nursing and Health Sciences, Monash University, Melbourne, VIC, Australia; 3 Faculty of Medicine, University of Colombo, Colombo, Sri Lanka

**Keywords:** access, admission, inclusive, selection, widening participation

The fulfillment of healthcare needs of society at large partially hinges on the shape and structure of the medical workforce. For example, a more diverse society requires a more diverse medical workforce (1) to serve the needs of the people. While this importance is noted, does it happen in reality? The challenges pertaining to the medical workforce are global but also simultaneously local, or “glocal” (2). In several countries, doctors are reluctant to work in remote and rural areas, but the influencing factors and the actions taken to resolve this issue may be contextual (3). Medical schools throughout the world need to apply different policies and methods to ensure diversity and inclusiveness in the student admission process (4). This special Research Topic was created to amplify and highlight considerations around widening participation in, and access to, medicine as a career.

In this Research Topic, there are 11 articles all focusing on this issue. From these, four broad themes have been extracted. These are: workforce planning and supply, career preference and pipeline, student selection and recruitment, and equality, diversity, and inclusion (EDI) in medical education.

Three articles (Lee and Shin; Campos et al.; Vasconez-Gonzalez et al.) discuss similar situations in South Korea, Brazil, and Ecuador, where the rapid expansion in the number of medical doctors has occurred without robust approaches to ensure the quality of medical training or appropriate policies and incentives for workforce distribution and retention, especially in underprivileged locations. The reasons proffered for this expansion include the low number of doctors in certain areas, especially rural, remote, or underprivileged areas. However, without proper planning for distribution and retention, graduates will search for better career opportunities and better earning and living conditions in major cities. The rushed expansion is likely to lower the quality medical training due to limited educational resources and teaching staff in newly opened medical schools or residency programs. Hence, a paradox exists whereby the increased number of medical doctors still cannot resolve the issue of short supply of doctors in certain areas.

With the oversupply of the medical workforce comes another challenge which relates to their career pipelines. One study by Dawood et al. highlighted trends among Pakistan's clinical undergraduate students in choosing their specialty programs. The authors observed a significant pivot from surgery to medicine as their chosen specialty training, which can be attributed to various internal and external factors, such as personal preferences, family matters, exposure to clinical rotations, and lack of mentorship. These factors should be taken into consideration when shaping the medical workforce to ensure the fulfillment of different specialties in all regions of a country. Mentoring and career support programs (5) from medical schools may be significant in helping students navigate their career preferences and pipelines while aligning with the needs of the country and society. Liu et al. surveyed Chinese high school students on the factors influencing their choice to pursue education in pediatrics. Among the 7,000 plus students surveyed, only a few (6.4%) showed an interest in pediatrics. These numbers may not reflect their actual decision in medical school, but they do emphasize the importance of promoting an accurate understanding of medicine and other specialties early on to spark their initial interest and willingness to pursue their chosen specialty as a career. Career days, hospital open days, mentoring, or shadowing opportunities are among the many activities that can enable more targeted recruitment of future doctors.

The third emergent theme of selection and recruitment of students is a starting point in the endeavor of shaping the medical workforce. For example, some studies have examined that students coming from rural areas are likelier to work in rural areas (6); hence, recruitment strategies should target more of those candidates from rural areas. Concerns related to selection are not only related to methods that demonstrate relatively good psychometric properties, for example, multiple mini interviews or MMIs (Szkwara et al.), but also to the admission criteria, which should be more inclusive of diverse students, including the disabled (Sayed). One of the articles in the Research Topic (Matos Sousa et al.) highlights the increased and sustained motivation of students entering through graduate-entry pathways. Policymakers should consider these findings when deciding on various routes of entry into medical schools. To create a diverse, inclusive, and relevant medical workforce, medical schools should not passively wait for candidates. Active recruitment can start as early as possible by exposing primary and secondary students to medicine to boost their interest in the field. An example of a relevant program is the “Doctor for a day” enrichment program specifically targeted toward underrepresented minority students (Coler et al.), which has been proven to increase their self-efficacy and interest in medicine.

Upholding diversity values should not stop at the admission process; ideally, the curriculum also needs to reflect equity, diversity and inclusion principles or EDI (7), which is the fourth theme. An article by Jerjes and Majeed (a) highlights how neurodiversity must be accounted for to provide a more inclusive training environment. Given that an increasing number of members of the population are suffering from certain types of neurodivergence, it is becoming more important for medical students to be trained in how to serve patients with neurodivergence and how the curriculum and medical school environment can cater to the need of neurodivergent trainees. In a second article by Jerjes and Majeed (b), the importance of curricula that more explicitly address non-visible disabilities is emphasized. It also argues that the assessment of the competencies required for doctors to care for patients with non-visible disabilities should be planned and implemented.

To conclude, ensuring medicine is accessible to everyone and creating a diverse medical workforce will require efforts on every spectrum, from workforce planning to the selection and recruitment process, followed by a curriculum that includes EDI and managing students' career pipelines so that not only is the quantity of doctors achieved but also the quality of the distribution process can be improved, including the distribution of doctors based on their specialties. The manuscripts in this volume help highlight these tricky problems, along with four aspects to help address the glocal issues at hand.
